# Synthesis and structure of 9-methyl-1,10-di­hydro­pyrazolo­[3,4-*a*]carbazole

**DOI:** 10.1107/S2056989026000502

**Published:** 2026-01-29

**Authors:** M. Sridharan, Aravazhi Amalan Thiruvalluvar, B. M. Rajesh

**Affiliations:** a Department of Chemistry, RV College of Engineering, Bangalore 560 059, Karnataka, India; b Principal (Retired), 63 Shanthi Nagar, 5th Street, Nanjikottai Road, Thanjavur 613 006, Tamilnadu, India; c Department of Physics, RV College of Engineering, Bangalore 560 059, Karnataka, India; University of Aberdeen, United Kingdom

**Keywords:** crystal structure, carbazole, Hirshfeld surface, N—H⋯N hy­dro­gen bonding, C—H⋯π contact

## Abstract

The title carbazole derivative, C_14_H_11_N_3_, was prepared by reacting 1-hy­droxy-8-methyl-9*H*-carbazole-2-carbaldehyde with hydrazine hydrate. In the solid state, the fused-ring system is slightly puckered, the dihedral angle between the planes of the outer rings being 2.24 (7)°. In the crystal, mol­ecules are linked by {N—H}_2_⋯N hy­dro­gen bonds to generate [010] chains, and weak C—H⋯π contacts consolidate the structure. A Hirshfeld surface analysis indicates that the most important contributions to the crystal packing are from H⋯H (43.1%), C⋯H/H⋯C (36.8%) and N⋯H/H⋯N (15.3%) inter­actions.

## Abstract 2

The extended structure of a pyrazolo­[3,4-*a*]carbazole features {N—H}_2_⋯N hy­dro­gen bonds, which generate [010] chains.

## 1. Chemical context

Carbazoles are tricyclic aromatic heterocycles that have attracted significant attention due to their presence in natural products and their wide-ranging biological activities. Synthetic methodologies to access carbazoles and their fused-ring derivatives include direct annulation, cyclization reactions and transition-metal catalysis (Knölker & Reddy, 2002 ▸). Linear and angular fused carbazoles, such as pyrido-, pyrazolo-, pyrimido- and pyridazinocarbazoles, possess pharmacological applications, including anti­tumour and anti-HIV activities, as well as an ability to act as DNA inter­calating agents (Kumar *et al.*, 2023 ▸; El-Essawy & Odah, 2024 ▸). Among these, pyrazolo-annulated heterocycles like pyrazolo­pyrido­pyrimidines stand out for their structural com­plexity, containing five N atoms and three fused rings, which combine the properties of pyrazole, pyridine and pyrimidine (Iorkula *et al.*, 2025 ▸). Beyond therapeutic applications, carbazole derivatives have emerged as versatile fluorescent chemosensors, enabling bioimaging of ionic species, reactive oxygen and sulfur species, biomacromolecules and microenvironments (Yin *et al.*, 2020 ▸). Synthetic efforts often employ 2,3,4,9-tetra­hydro­carbazol-1-ones as precursors, which provide easily accessible inter­mediates for the construction of diverse heteroannulated carbazoles (*e.g.* Suvarna *et al.*, 2024 ▸). In particular, pyrazolo­[3,4-*a*]carbazoles bridge the gap between natural carbazole alkaloids and synthetic medicinal chemistry, offering a scaffold of broad medical importance in oncology, infectious disease and neurology (Ramoba *et al.*, 2025 ▸; Menezes & Bhat, 2025 ▸). As part of our studies in this area, we now describe the synthesis and structure of the title com­pound 9-methyl-1,10-di­hydro­pyrazolo­[3,4-*a*]carbazole, (**I**).

## 2. Structural commentary

**Figure 1:**
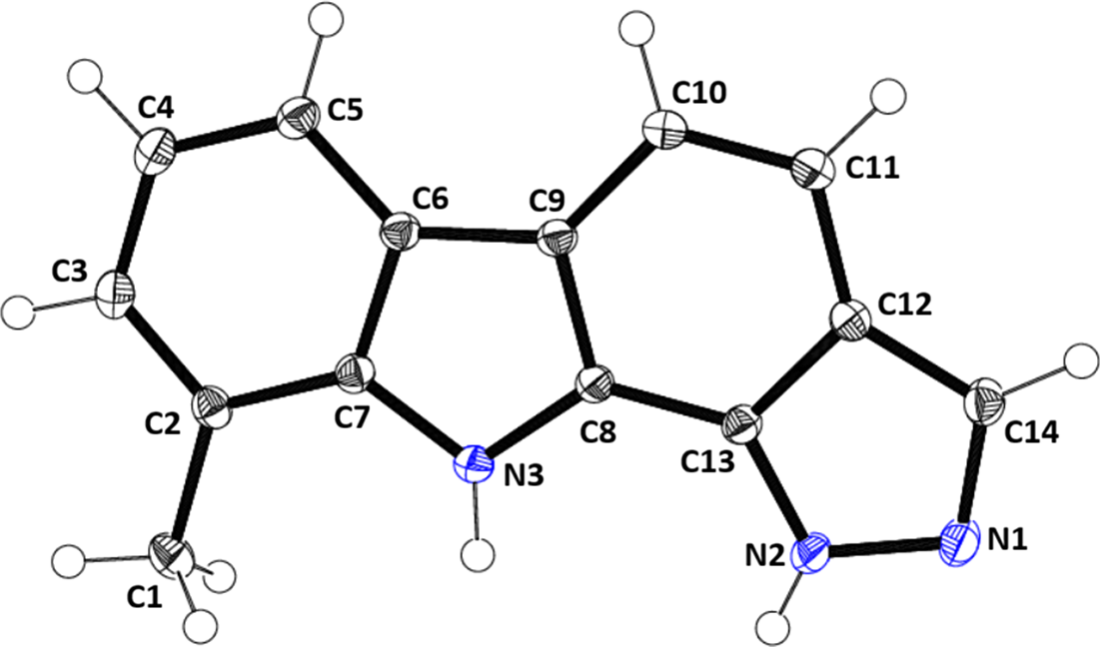
The mol­ecular structure of (**I**), showing displacement ellipsoids drawn at the 50% probability level.

In the solid state, com­pound (**I**) (Fig. 1 ▸) is slightly puckered, the dihedral angle between the outer C2–C7 and N1/N2/C13/C12/C14 rings being 2.24 (7)°. The dihedral angle between the inner C6–C9/N3 and C8–C13 rings is 1.79 (7)°. Alternately, the mol­ecule may be regarded as almost planar, the r.m.s. deviation from planarity for all the C and N atoms being 0.022 Å. Significant bond lengths and angles include C6—C9 [1.447 (2) Å], C11—C12 [1.425 (2) Å], N1—N2 [1.3667 (19) Å], N3—C8—C13 [130.82 (13)°], C13—N2—N1 [111.40 (12)°] and N2—N1—C14 [105.60 (12)°].
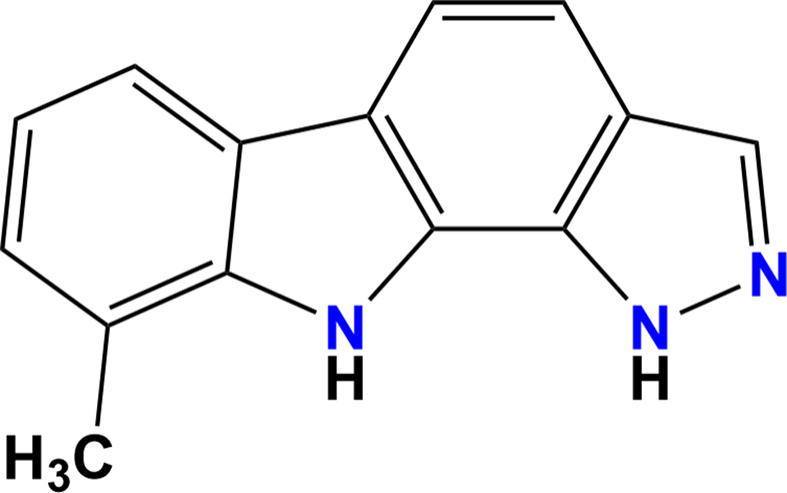


## 3. Supra­molecular features

**Figure 2:**
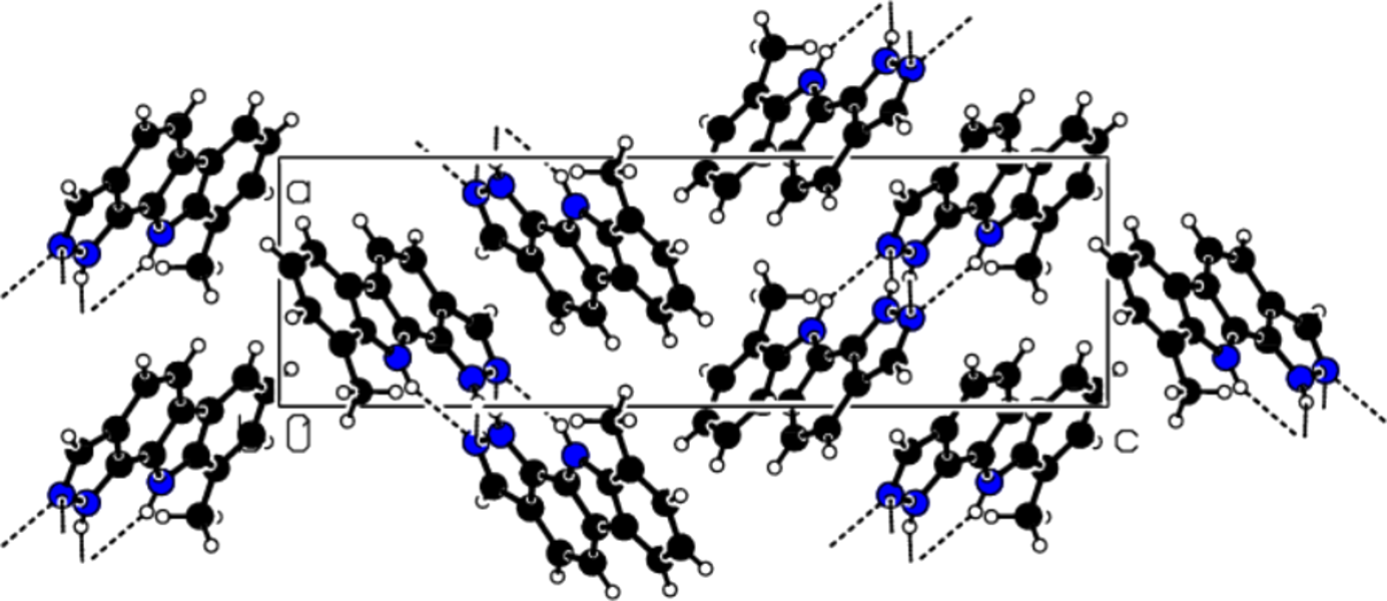
Partial packing view of (**I**), viewed down the *b*-axis direction, showing the hy­dro­gen bonds. Black dashed lines represent N—H⋯N hy­dro­gen bonds.

**Figure 3:**
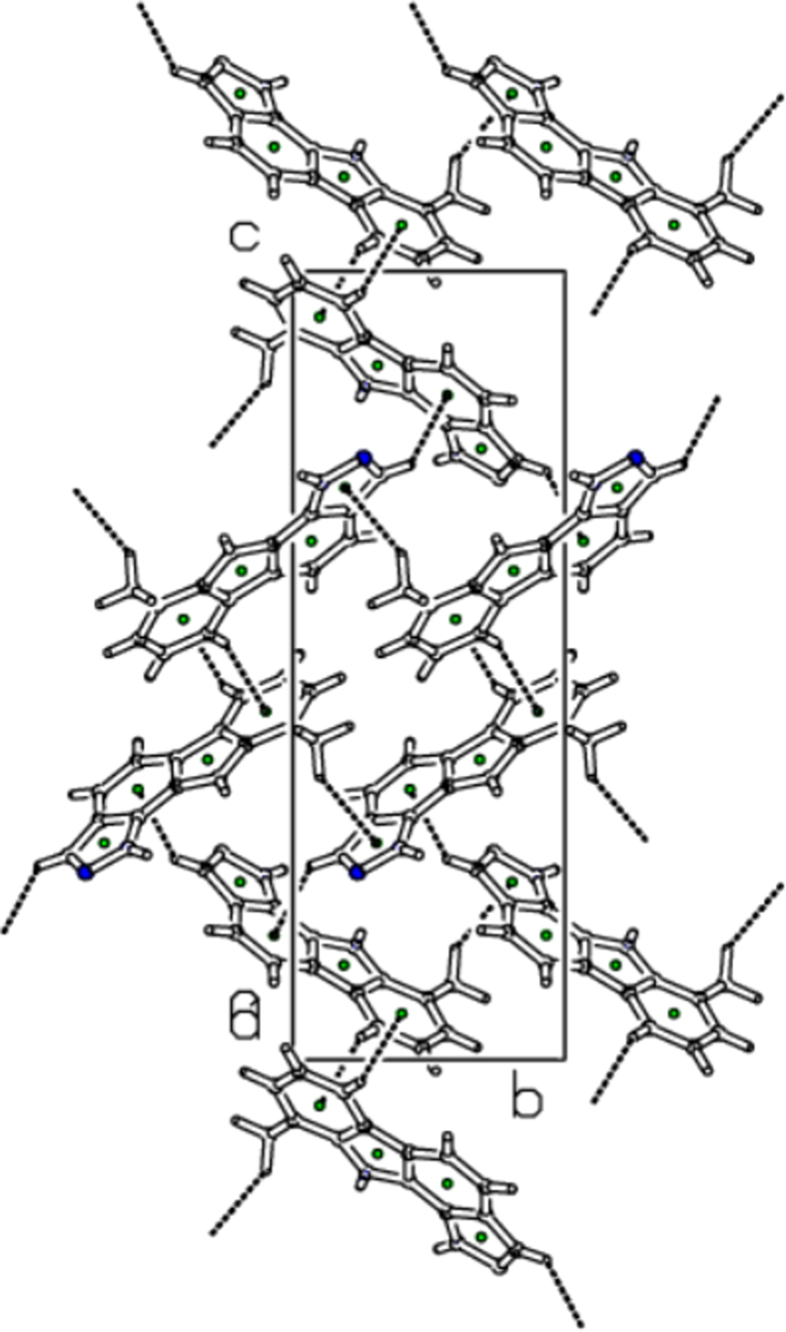
Straw-style packing view of (**I**), viewed down the *a*-axis direction, showing the C—H⋯π contacts. Centroids are given as green spheres and black dashed lines are H⋯π contacts.

In the extended structure of (**I**), the mol­ecules are linked by N—H⋯N hy­dro­gen bonds, with atom N1 accepting two such bonds from both N2—H2 and N3—H3*A* (Table 1 ▸ and Fig. 2 ▸). This generates an [010] chain, with adjacent mol­ecules in the chain related by a 2_1_ screw axis. The packing also exhibits three weak C—H⋯π inter­actions that connect parallel chains (Fig. 3 ▸ and Table 1 ▸). The mol­ecules exhibit some apparent offset π–π stacking inter­actions with an inter­planar spacing of 3.491 Å between mol­ecules related by a translation along the *b* axis. However, a qu­anti­tative analysis of these inter­actions (see *Hirshfeld surface analysis* section below) suggests that they make a very minor contribution to the overall packing of (**I**).

## 4. Database survey

A search of the Cambridge Structural Database (CSD, Version 6.01, updated to November 2025; Groom *et al.*, 2016 ▸) using the core structure of (**I**) gave one hit, namely, 3,9-dimethyl-1,10-di­hydro­pyrazolo­[3,4-*a*]carbazole (CSD refcode TIGPIJ; Martin *et al.*, 2007 ▸), in which a methyl group occurs additionally at the 3-position [atom C14 in (**I**)]. The only other com­pound sharing the same core tetra­cyclic structure as the title com­pound is 7-methyl-1-phenyl-1,10-di­hydro­pyrazolo­[3,4-*a*]carbazole (CSD refcode ZIJGIK), featuring a phenyl substituent at the pyrazole N atom [N2 in (**I**)] and a methyl group at the 7-position [C4 in (**I**)] (Archana *et al.*, 2013 ▸).

## 5. Hirshfeld surface (HS) and 2D fingerprint plots

**Figure 4:**
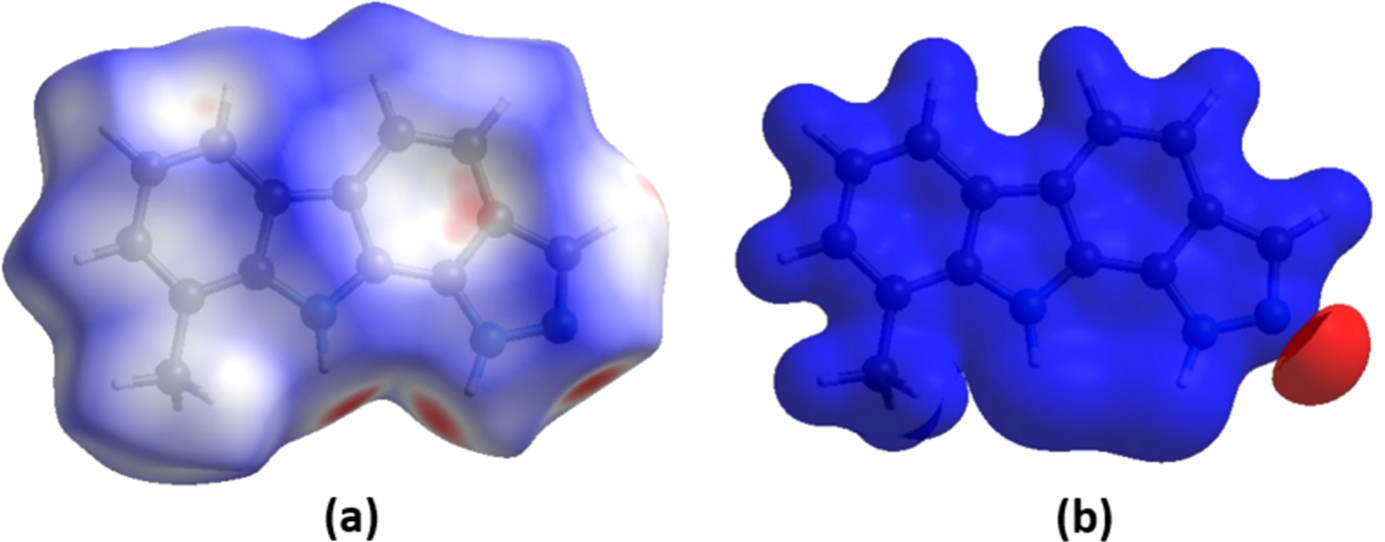
(*a*) View of the three-dimensional Hirshfeld surface of (**I**), plotted over *d*_norm_ in the range from −0.39 to 1.20 a.u. (*b*) View of the three-dimensional electrostatic potential surface of (**I**) plotted over the range from −0.05 to 0.05 a.u., using the STO-3G basis set at the Hartree–Fock method of theory.

*CrystalExplorer* (Version 21.5; Spackman *et al.*, 2021 ▸) was used to investigate and visualize further the inter­molecular inter­actions of (**I**). The HS plotted over *d*_norm_ in the range from −0.39 to 1.20 a.u. is shown in Fig. 4 ▸(*a*). The electrostatic potential surface using the STO-3G basis set at the Hartree–Fock level of theory and mapped on the Hirshfeld surface over the range from −0.05 to 0.05 a.u. clearly shows the positions of the close inter­molecular contacts in the com­pound [Fig. 4 ▸(*b*)]. The positive electrostatic potential (blue area) over the surface indicates hy­dro­gen-donor potential, whereas the negative (red area) represents the hy­dro­gen-bond acceptors.

**Figure 5:**
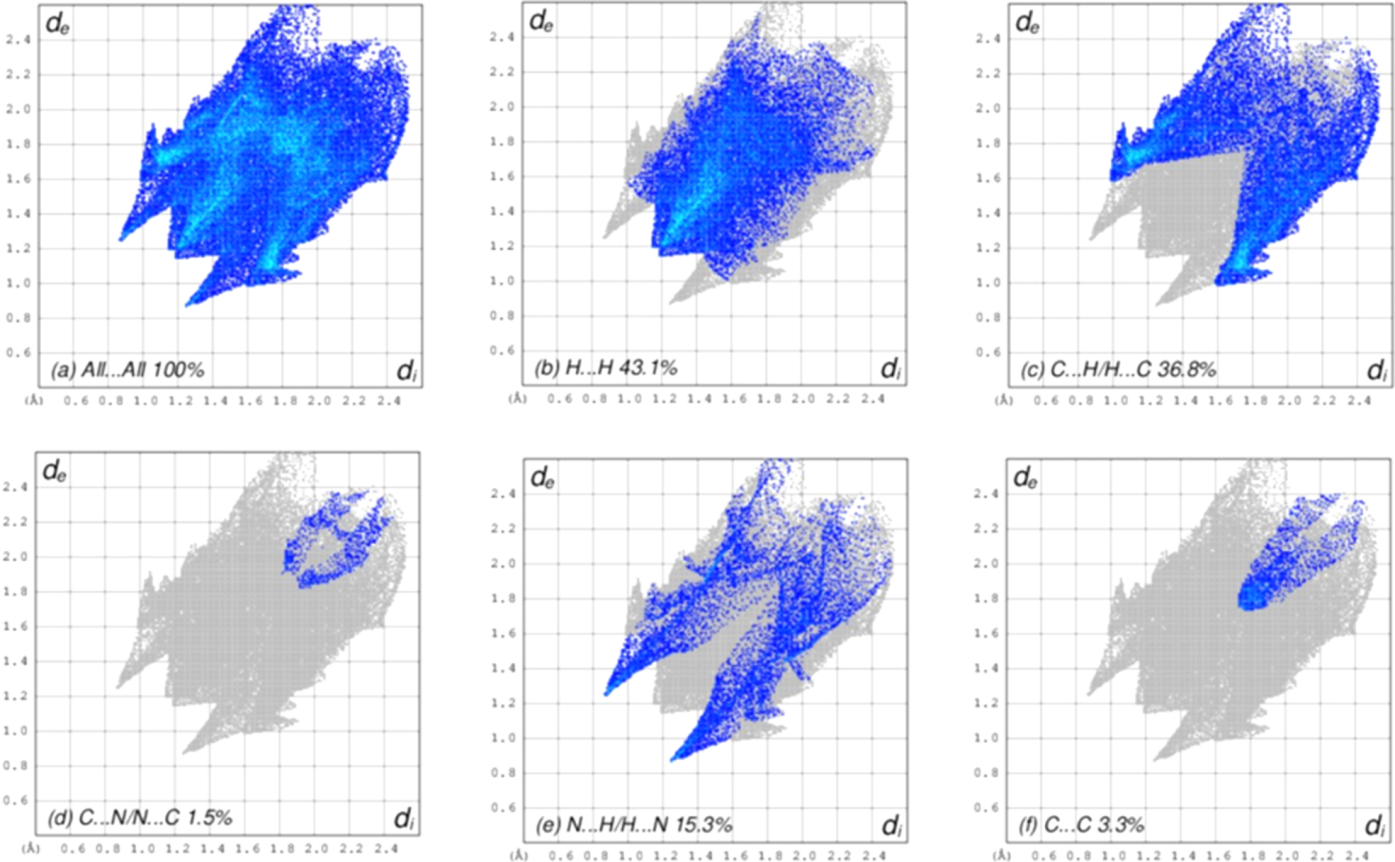
Two-dimensional fingerprint plots for (**I**), showing (*a*) all inter­actions, and delineated into (*b*) H⋯H, (*c*) C⋯H/H⋯C, (*d*) C⋯N/N⋯C, (*e*) N⋯H/H⋯N and (*f*) C⋯C inter­actions. The *d*_i_ and *d*_e_ values are the closest inter­nal and external distances (in Å) from given points on the Hirshfeld surface.

The overall two-dimensional fingerprint plot is shown in Fig. 5 ▸(*a*), while those delineated into H⋯H, C⋯H/H⋯C, C⋯N/N⋯C, N⋯H/H⋯N and C⋯C contacts are illustrated in Figs. 5 ▸(*b*)–5(*f*), respectively, together with their relative contributions to the Hirshfeld surface. The most significant inter­action type is H⋯H, contributing 43.1% to the Hirshfeld surface, which is reflected in Fig. 5 ▸(*b*) as widely scattered points of high density due to the large hy­dro­gen content of the mol­ecule. In the presence of C⋯H inter­actions, the pair of characteristic wings in the fingerprint plot is delineated into C⋯H/H⋯C contacts [36.8% contribution to the HS; Fig. 5 ▸(*c*)]. The C⋯N/N⋯C contacts contribute only 1.5% [Fig. 5 ▸(*d*)] and the N⋯H/H⋯N contacts contribute 15.3% [Fig. 5 ▸(*e*)]. Finally, the C⋯C contacts [Fig. 5 ▸(*f*)] contribute only 3.3%. The packing of (**I**) is thus dominated by van der Waals inter­actions, augmented by N—H⋯N hy­dro­gen bonds and some C—H⋯π inter­actions, while π–π inter­actions play only a very minor role, despite the planar nature of the individual mol­ecules.

For a DFT and mol­ecular docking study of (**I**), see the supporting information.

## 6. Synthesis and crystallization

**Figure 6:**
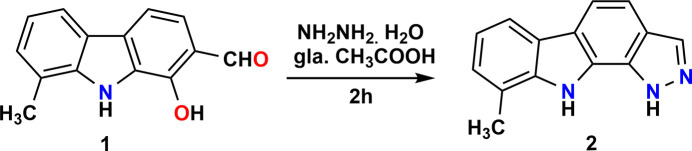
The synthesis of (**I**).

A solution of 1-hy­droxy-8-methyl-9*H*-carbazole-2-carbaldehyde (0.001 mol) in glacial acetic acid (20 ml) was treated with hydrazine hydrate (0.1 ml, 0.002 mol) under continuous stirring. The reaction mixture was subjected to reflux in an oil bath for 2 h, and the progress of the transformation was monitored periodically by thin-layer chromatography (TLC) using petroleum ether–ethyl acetate (8:2 *v*/*v*) as the mobile phase. Upon com­pletion, the hot reaction mixture was poured onto crushed ice, resulting in the immediate precipitation of a yellow solid. The solid was collected by vacuum filtration, washed thoroughly with distilled water to remove residual acetic acid and air-dried. The crude product was further purified by column chromatography over silica gel, employing petroleum ether–ethyl acetate (90:10 *v*/*v*) as the eluent. This afforded the title com­pound as a yellow crystalline solid (Fig. 6 ▸). Yellow prisms of (**I**) were recrystallized from ethanol solution.

Pale-yellow solid (0.191 g, 86%); m.p. 474–476 K; IR: ν_max_ 3393, 2919, 1619, 1570, 1480, 1228, 1056, 857 cm^−1^. ^1^H NMR: δ 12.48 (*b s*, 1H, pyrazole –NH), 11.16 (*s*, 1H, N10-H), 8.16 (*s*, 1H, C3-H), 7.96 (*d*, 1H, C6-H, *J* = 7.56 Hz), 7.82 (*d*, 1H, C4-H, *J* = 8.44 Hz), 7.48 (*d*, 1H, C5-H, *J* = 8.44 Hz), 7.20 (*d*, 1H, C8-H, *J* = 6.88 Hz), 7.12 (*t*, 1H, C7-H, *J* = 7.60 Hz), 2.48 (*s*, 3H, C9-CH_3_). MS: *m*/*z* (%) 221 (*M*^+^ = 100). Analysis calculated (%) for C_14_H_11_N_3_: C 76.00, H 5.01, N 18.99; found: C 75.89, H 4.92, N 18.76.

## 7. Refinement

Crystal data, data collection and structure refinement details are summarized in Table 2 ▸. Atoms H2 and H3*A* bonded to N2 and N3 were located in a difference Fourier map and refined isotropically with *U*_iso_(H) = 1.2*U*_eq_(N). All the other H atoms were placed in calculated positions and were refined with *U*_iso_(H) = 1.2*U*_eq_(C) or 1.5*U*_eq_(methyl C).

## Supplementary Material

Crystal structure: contains datablock(s) I, global. DOI: 10.1107/S2056989026000502/hb8187sup1.cif

Structure factors: contains datablock(s) I. DOI: 10.1107/S2056989026000502/hb8187Isup2.hkl

Supporting information file. DOI: 10.1107/S2056989026000502/hb8187Isup3.cdx

Density functional theory calculations and Molecular docking studies. DOI: 10.1107/S2056989026000502/hb8187sup4.pdf

Supporting information file. DOI: 10.1107/S2056989026000502/hb8187Isup5.cml

CCDC reference: 2524177

Additional supporting information:  crystallographic information; 3D view; checkCIF report

## Figures and Tables

**Table 1:** Hydrogen-bond geometry (Å, °) *Cg*1, *Cg*3 and *Cg*4 are the centroids of the N1/N2/C13/C12/C14, C2–C7 and C8–C13 rings, respectively.

*D*—H⋯*A*	*D*—H	H⋯*A*	*D*⋯*A*	*D*—H⋯*A*
N2—H2⋯N1^i^	0.80 (2)	2.37 (2)	3.097 (2)	150.9 (19)
N3—H3*A*⋯N1^i^	0.88 (2)	2.24 (2)	3.050 (2)	152.8 (17)
C1—H1*B*⋯*Cg*1^ii^	0.98	2.74	3.504 (3)	135
C5—H5⋯*Cg*3^iii^	0.95	2.57	3.408 (3)	147
C14—H14⋯*Cg*4^iv^	0.95	2.47	3.264 (3)	142

Symmetry codes: (i) 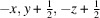; (ii) 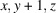; (iii) 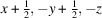; (iv) 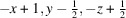.

**Table 2:** Experimental details

Crystal data	
Chemical formula	C_14_H_11_N_3_
*M*_r_	221.26
Crystal system, space group	Orthorhombic, *P*2_1_2_1_2_1_
Temperature (K)	100
*a*, *b*, *c* (Å)	6.570 (4), 7.541 (5), 21.854 (14)
*V* (Å^3^)	1082.8 (12)
*Z*	4
Radiation type	Mo *K*α
μ (mm^−1^)	0.08
Crystal size (mm)	0.45 × 0.28 × 0.25

Data collection	
Diffractometer	Bruker SMART APEX CCD
Absorption correction	Multi-scan (*SADABS2016*; Krause *et al.*, 2015 ▸)
*T*_min_, *T*_max_	0.714, 0.746
No. of measured, independent and observed [*I* > 2σ(*I*)] reflections	13749, 3671, 3470
*R*_int_	0.024
(sin θ/λ)_max_ (Å^−1^)	0.752

Refinement	
*R*[*F*^2^ > 2σ(*F*^2^)], *wR*(*F*^2^), *S*	0.037, 0.098, 1.05
No. of reflections	3671
No. of parameters	161
H-atom treatment	H atoms treated by a mixture of independent and constrained refinement
Δρ_max_, Δρ_min_ (e Å^−3^)	0.37, −0.25
Absolute structure	Flack *x* determined using 1366 quotients [(*I*^+^)−(*I*^−^)]/[(*I*^+^)+(*I*^−^)] (Parsons *et al.*, 2013 ▸)
Absolute structure parameter	0.5 (6)

Computer programs: *APEX2* (Bruker, 2007 ▸), *SAINT* (Bruker, 2025 ▸), *SHELXS* (Sheldrick, 2008 ▸), *SHELXL2025* (Sheldrick, 2015 ▸), *PLATON* (Spek, 2020 ▸) and *publCIF* (Westrip, 2010 ▸).

## supplementary crystallographic information

### 9-Methyl-1,10-dihydropyrazolo[3,4-a]carbazole Crystal data

**Table:**

C_14_H_11_N_3_	*D*_x_ = 1.357 Mg m^−^^3^
*M**_r_* = 221.26	Melting point: 475(1) K
Orthorhombic, *P*2_1_2_1_2_1_	Mo *K*α radiation, λ = 0.71073 Å
*a* = 6.570 (4) Å	Cell parameters from 5839 reflections
*b* = 7.541 (5) Å	θ = 2.9–32.2°
*c* = 21.854 (14) Å	µ = 0.08 mm^−^^1^
*V* = 1082.8 (12) Å^3^	*T* = 100 K
*Z* = 4	Prism, yellow
*F*(000) = 464	0.45 × 0.28 × 0.25 mm

### 9-Methyl-1,10-dihydropyrazolo[3,4-a]carbazole Data collection

**Table:**

Bruker SMART APEX CCD diffractometer	3671 independent reflections
Radiation source: fine-focus sealed tube	3470 reflections with *I* > 2σ(*I*)
Graphite monochromator	*R*_int_ = 0.024
ω scans	θ_max_ = 32.3°, θ_min_ = 1.9°
Absorption correction: multi-scan (SADABS2016; Krause *et al.*, 2015)	*h* = −9→9
*T*_min_ = 0.714, *T*_max_ = 0.746	*k* = −11→10
13749 measured reflections	*l* = −32→31

### 9-Methyl-1,10-dihydropyrazolo[3,4-a]carbazole Refinement

**Table:**

Refinement on *F*^2^	Secondary atom site location: difference Fourier map
Least-squares matrix: full	Hydrogen site location: mixed
*R*[*F*^2^ > 2σ(*F*^2^)] = 0.037	H atoms treated by a mixture of independent and constrained refinement
*wR*(*F*^2^) = 0.098	*w* = 1/[σ^2^(*F*_o_^2^) + (0.0582*P*)^2^ + 0.1706*P*] where *P* = (*F*_o_^2^ + 2*F*_c_^2^)/3
*S* = 1.05	(Δ/σ)_max_ < 0.001
3671 reflections	Δρ_max_ = 0.37 e Å^−^^3^
161 parameters	Δρ_min_ = −0.25 e Å^−^^3^
0 restraints	Absolute structure: Flack *x* determined using 1366 quotients [(I+)-(I-)]/[(I+)+(I-)] (Parsons *et al.*, 2013)
Primary atom site location: structure-invariant direct methods	Absolute structure parameter: 0.5 (6)

### 9-Methyl-1,10-dihydropyrazolo[3,4-a]carbazole Special details

**Table:**

Geometry. All esds (except the esd in the dihedral angle between two l.s. planes) are estimated using the full covariance matrix. The cell esds are taken into account individually in the estimation of esds in distances, angles and torsion angles; correlations between esds in cell parameters are only used when they are defined by crystal symmetry. An approximate (isotropic) treatment of cell esds is used for estimating esds involving l.s. planes.

### 9-Methyl-1,10-dihydropyrazolo[3,4-a]carbazole Fractional atomic coordinates and isotropic or equivalent isotropic displacement parameters (Å^2^)

**Table:**

	*x*	*y*	*z*	*U*_iso_*/*U*_eq_	
C1	0.0602 (2)	0.5882 (2)	0.09586 (7)	0.0194 (3)	
H1A	0.056164	0.706817	0.077613	0.029*	
H1B	0.057787	0.598395	0.140561	0.029*	
H1C	−0.058266	0.519946	0.082146	0.029*	
C2	0.2520 (2)	0.49503 (19)	0.07631 (6)	0.0153 (2)	
C3	0.3868 (2)	0.56427 (19)	0.03354 (6)	0.0176 (3)	
H3	0.358181	0.676651	0.015884	0.021*	
C4	0.5644 (2)	0.47378 (19)	0.01542 (7)	0.0181 (3)	
H4	0.651095	0.525425	−0.014452	0.022*	
C5	0.6148 (2)	0.31070 (19)	0.04042 (6)	0.0163 (3)	
H5	0.735680	0.250830	0.028457	0.020*	
C6	0.4828 (2)	0.23636 (18)	0.08385 (6)	0.0139 (2)	
C7	0.3035 (2)	0.32869 (18)	0.10041 (6)	0.0141 (2)	
C8	0.3057 (2)	0.07438 (18)	0.15330 (6)	0.0135 (2)	
C9	0.4842 (2)	0.07220 (17)	0.11814 (6)	0.0138 (2)	
C10	0.6257 (2)	−0.07029 (19)	0.12205 (6)	0.0162 (2)	
H10	0.745464	−0.068954	0.097703	0.019*	
C11	0.5891 (2)	−0.21041 (18)	0.16118 (6)	0.0165 (3)	
H11	0.682735	−0.306001	0.164139	0.020*	
C12	0.4079 (2)	−0.20876 (18)	0.19703 (6)	0.0144 (2)	
C13	0.26791 (19)	−0.06831 (18)	0.19293 (6)	0.0140 (2)	
C14	0.3203 (2)	−0.32329 (19)	0.24136 (6)	0.0173 (3)	
H14	0.379954	−0.431679	0.254346	0.021*	
N1	0.14434 (18)	−0.26063 (18)	0.26256 (6)	0.0195 (2)	
N2	0.11285 (18)	−0.10440 (17)	0.23232 (6)	0.0173 (2)	
N3	0.19676 (18)	0.22779 (16)	0.14285 (5)	0.0152 (2)	
H2	0.017 (3)	−0.042 (3)	0.2393 (9)	0.018*	
H3A	0.077 (3)	0.250 (3)	0.1598 (9)	0.018*	

### 9-Methyl-1,10-dihydropyrazolo[3,4-a]carbazole Atomic displacement parameters (Å^2^)

**Table:**

	*U*^11^	*U*^22^	*U*^33^	*U*^12^	*U*^13^	*U*^23^
C1	0.0191 (6)	0.0179 (6)	0.0212 (6)	0.0043 (5)	−0.0022 (5)	0.0003 (5)
C2	0.0175 (6)	0.0139 (6)	0.0146 (5)	0.0009 (5)	−0.0018 (4)	−0.0007 (5)
C3	0.0221 (6)	0.0143 (6)	0.0163 (6)	−0.0018 (5)	−0.0012 (5)	0.0014 (5)
C4	0.0209 (6)	0.0175 (6)	0.0160 (6)	−0.0042 (5)	0.0013 (5)	0.0017 (5)
C5	0.0174 (6)	0.0176 (6)	0.0140 (5)	−0.0013 (5)	0.0017 (5)	0.0001 (5)
C6	0.0145 (5)	0.0144 (5)	0.0129 (5)	0.0000 (4)	0.0009 (4)	−0.0006 (4)
C7	0.0155 (5)	0.0146 (5)	0.0121 (5)	−0.0001 (5)	0.0001 (4)	0.0001 (4)
C8	0.0129 (5)	0.0142 (5)	0.0133 (5)	0.0008 (5)	0.0006 (4)	0.0012 (4)
C9	0.0138 (5)	0.0149 (5)	0.0126 (5)	0.0005 (5)	0.0015 (4)	−0.0003 (5)
C10	0.0150 (5)	0.0166 (6)	0.0168 (6)	0.0019 (5)	0.0025 (5)	−0.0004 (5)
C11	0.0160 (6)	0.0156 (6)	0.0179 (6)	0.0019 (5)	0.0010 (5)	−0.0006 (5)
C12	0.0143 (6)	0.0140 (5)	0.0149 (5)	0.0003 (4)	−0.0008 (4)	−0.0004 (5)
C13	0.0128 (5)	0.0156 (6)	0.0137 (5)	−0.0003 (4)	0.0005 (4)	0.0012 (5)
C14	0.0155 (6)	0.0173 (6)	0.0191 (6)	0.0000 (5)	−0.0012 (5)	0.0042 (5)
N1	0.0170 (5)	0.0205 (6)	0.0210 (6)	−0.0008 (5)	0.0013 (4)	0.0074 (5)
N2	0.0143 (5)	0.0188 (6)	0.0189 (5)	0.0016 (4)	0.0039 (4)	0.0058 (5)
N3	0.0141 (5)	0.0156 (5)	0.0158 (5)	0.0024 (4)	0.0024 (4)	0.0017 (4)

### 9-Methyl-1,10-dihydropyrazolo[3,4-a]carbazole Geometric parameters (Å, º)

**Table:**

C1—C2	1.505 (2)	C8—C9	1.4023 (19)
C1—H1A	0.9800	C8—C13	1.403 (2)
C1—H1B	0.9800	C9—C10	1.424 (2)
C1—H1C	0.9800	C10—C11	1.380 (2)
C2—C3	1.390 (2)	C10—H10	0.9500
C2—C7	1.402 (2)	C11—C12	1.425 (2)
C3—C4	1.408 (2)	C11—H11	0.9500
C3—H3	0.9500	C12—C13	1.4058 (19)
C4—C5	1.386 (2)	C12—C14	1.420 (2)
C4—H4	0.9500	C13—N2	1.3612 (18)
C5—C6	1.4027 (19)	C14—N1	1.3319 (19)
C5—H5	0.9500	C14—H14	0.9500
C6—C7	1.415 (2)	N1—N2	1.3667 (19)
C6—C9	1.447 (2)	N2—H2	0.80 (2)
C7—N3	1.3897 (18)	N3—H3A	0.88 (2)
C8—N3	1.3794 (18)		
			
C2—C1—H1A	109.5	C9—C8—C13	118.49 (12)
C2—C1—H1B	109.5	C8—C9—C10	121.49 (13)
H1A—C1—H1B	109.5	C8—C9—C6	105.57 (11)
C2—C1—H1C	109.5	C10—C9—C6	132.91 (12)
H1A—C1—H1C	109.5	C11—C10—C9	120.08 (13)
H1B—C1—H1C	109.5	C11—C10—H10	120.0
C3—C2—C7	115.78 (13)	C9—C10—H10	120.0
C3—C2—C1	123.33 (13)	C10—C11—C12	118.65 (12)
C7—C2—C1	120.88 (13)	C10—C11—H11	120.7
C2—C3—C4	122.35 (14)	C12—C11—H11	120.7
C2—C3—H3	118.8	C13—C12—C14	103.67 (12)
C4—C3—H3	118.8	C13—C12—C11	121.21 (12)
C5—C4—C3	121.15 (13)	C14—C12—C11	135.12 (13)
C5—C4—H4	119.4	N2—C13—C8	132.52 (13)
C3—C4—H4	119.4	N2—C13—C12	107.39 (12)
C4—C5—C6	118.25 (13)	C8—C13—C12	120.09 (12)
C4—C5—H5	120.9	N1—C14—C12	111.94 (13)
C6—C5—H5	120.9	N1—C14—H14	124.0
C5—C6—C7	119.42 (13)	C12—C14—H14	124.0
C5—C6—C9	133.50 (13)	C14—N1—N2	105.60 (12)
C7—C6—C9	107.07 (11)	C13—N2—N1	111.40 (12)
N3—C7—C2	128.21 (13)	C13—N2—H2	126.2 (14)
N3—C7—C6	108.76 (12)	N1—N2—H2	122.3 (14)
C2—C7—C6	123.03 (13)	C8—N3—C7	107.93 (12)
N3—C8—C9	110.67 (12)	C8—N3—H3A	123.4 (13)
N3—C8—C13	130.82 (13)	C7—N3—H3A	128.6 (13)
			
C7—C2—C3—C4	−0.1 (2)	C6—C9—C10—C11	177.71 (14)
C1—C2—C3—C4	−179.28 (13)	C9—C10—C11—C12	0.1 (2)
C2—C3—C4—C5	−1.0 (2)	C10—C11—C12—C13	0.2 (2)
C3—C4—C5—C6	0.8 (2)	C10—C11—C12—C14	−179.27 (15)
C4—C5—C6—C7	0.31 (19)	N3—C8—C13—N2	1.7 (3)
C4—C5—C6—C9	179.06 (14)	C9—C8—C13—N2	179.72 (14)
C3—C2—C7—N3	−179.06 (14)	N3—C8—C13—C12	−177.62 (14)
C1—C2—C7—N3	0.2 (2)	C9—C8—C13—C12	0.36 (19)
C3—C2—C7—C6	1.26 (19)	C14—C12—C13—N2	−0.32 (15)
C1—C2—C7—C6	−179.52 (13)	C11—C12—C13—N2	−179.92 (13)
C5—C6—C7—N3	178.86 (12)	C14—C12—C13—C8	179.19 (12)
C9—C6—C7—N3	−0.19 (15)	C11—C12—C13—C8	−0.4 (2)
C5—C6—C7—C2	−1.4 (2)	C13—C12—C14—N1	0.00 (16)
C9—C6—C7—C2	179.54 (12)	C11—C12—C14—N1	179.52 (15)
N3—C8—C9—C10	178.28 (12)	C12—C14—N1—N2	0.32 (16)
C13—C8—C9—C10	−0.1 (2)	C8—C13—N2—N1	−178.88 (14)
N3—C8—C9—C6	−0.09 (15)	C12—C13—N2—N1	0.54 (16)
C13—C8—C9—C6	−178.45 (12)	C14—N1—N2—C13	−0.53 (16)
C5—C6—C9—C8	−178.69 (15)	C9—C8—N3—C7	−0.02 (15)
C7—C6—C9—C8	0.17 (14)	C13—C8—N3—C7	178.07 (14)
C5—C6—C9—C10	3.2 (3)	C2—C7—N3—C8	−179.58 (13)
C7—C6—C9—C10	−177.93 (14)	C6—C7—N3—C8	0.13 (15)
C8—C9—C10—C11	−0.1 (2)		

### 9-Methyl-1,10-dihydropyrazolo[3,4-a]carbazole Hydrogen-bond geometry (Å, º)

Cg1, Cg3 and Cg4 are the centroids of the N1/N2/C13/C12/C14, C2–C7 and C8–C13 rings respectively.

**Table:**

*D*—H···*A*	*D*—H	H···*A*	*D*···*A*	*D*—H···*A*
N2—H2···N1^i^	0.80 (2)	2.37 (2)	3.097 (2)	150.9 (19)
N3—H3*A*···N1^i^	0.88 (2)	2.24 (2)	3.050 (2)	152.8 (17)
C1—H1*B*···*Cg*1^ii^	0.98	2.74	3.504 (3)	135
C5—H5···*Cg*3^iii^	0.95	2.57	3.408 (3)	147
C14—H14···*Cg*4^iv^	0.95	2.47	3.264 (3)	142

Symmetry codes: (i) −*x*, *y*+1/2, −*z*+1/2; (ii) *x*, *y*+1, *z*; (iii) *x*+1/2, −*y*+1/2, −*z*; (iv) −*x*+1, *y*−1/2, −*z*+1/2.
