# Supplementary material for: Synthesis and structure of 9-methyl-1,10-di­hydro­pyrazolo­[3,4-a]carbazole
Source: Acta Crystallogr E Crystallogr Commun. 2026 Jan 29;82(Pt 2):231–4. doi: 10.1107/S2056989026000502 (PMC12874232; doi:10.1107/S2056989026000502)
Supplement: Supplementary file 4 [file e-82-00231-sup4.pdf]

STRUCTURE REPORTS

Volume 82 (2026)

Supporting information for article:

Synthesis and structure of 9-methyl-1,10-dihydropyrazolo[3,4-*a*]carbazole

M. Sridharan, Aravazhi Amalan Thiruvalluvar and B. M. Rajesh

hb8187_supplementary material

S1. Density functional theory calculations:

Density functional theory (DFT) calculations were performed to better understand the experimental data of the studied compound. To understand the frontier molecular orbital energies of the titled compound $C_{14}H_{11}N_3$, the structure was optimized with Gaussian 16W, Revision C.01, Version 1.1 (Frisch *et al.*, 2019) package using the DFT method using a B3LYP functional with a 6–31G(d,p) basis set based on the single-crystal X-ray diffraction structure data. The results were visualized using GaussView 6.1.1 (Dennington *et al.*, 2019). Other molecular properties such as highest occupied molecular orbital (HOMO) – lowest unoccupied molecular orbital (LUMO) energies, and Mulliken charge analysis were also obtained. Vibrational frequency calculations were conducted to get IR and Raman frequencies and confirm the structure as a true energy minimum as confirmed by the absence of imaginary frequencies (spectra not shown).

The optimized structure and its atom numbering are displayed in **Figure S1 (a)** and the Mulliken charges in **Figure S1 (b)**. The optimized geometry (in gaseous state) is highly correlated with the single-crystal X-ray data (in solid state) of the title compound with a correlation coefficient of 0.99 and 0.99 for the bond lengths (**Figure S2 (a)**) and for the bond angles (**Figure S2 (b)**), respectively. The overall energy minimum is –704.4 Hartree with a dipole moment (μ) of 2.71 Debye, and a polarizability α of 169.8 a.u., values that agree with expectations for a carbazole molecule such as (I).

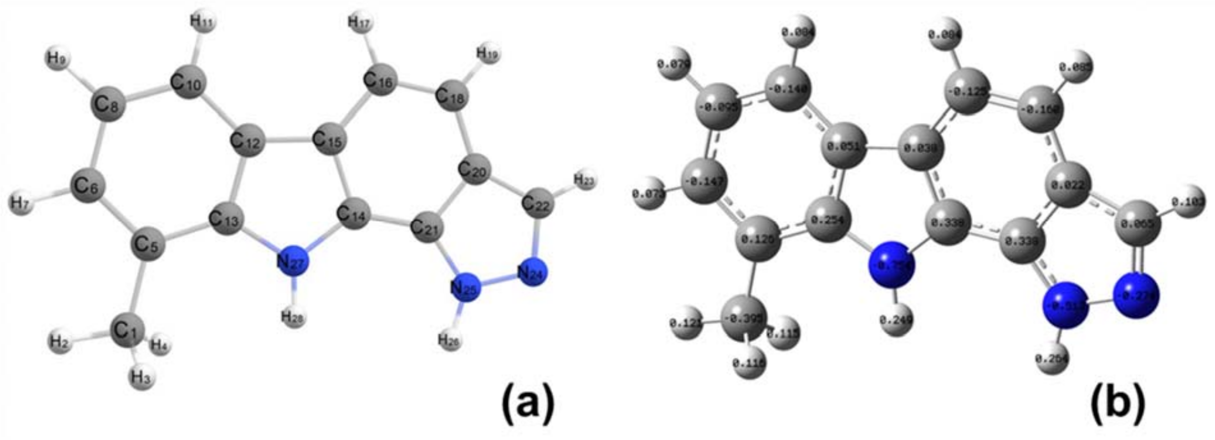

Figure S1

Optimized structure of the titled compound (a) with the Mulliken charges (b).

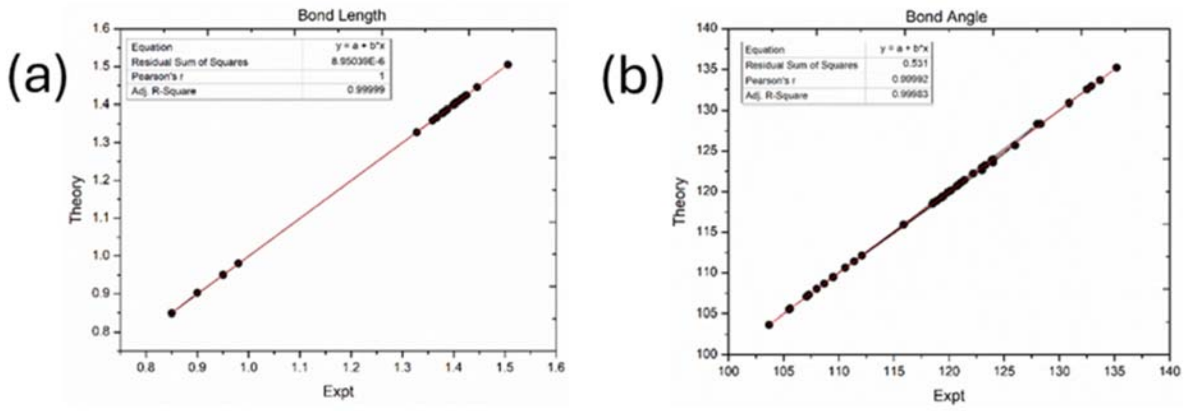

Figure S2

The correlation diagram for the optimized geometry with the single-crystal X-ray data of the title compound for the bond lengths (a) and bond angles (b).

Frontier molecular orbital (FMO) analysis was performed to evaluate the electronic characteristics of the title compound (I). The electron transition from the HOMO to the LUMO energy level is shown in **Figure S3**. The calculated Energy gap Eg (ΔE = 4.60 eV), the ionization potential (IP = –5.31 eV), electron affinity (EA = –0.71 eV), the chemical potential (μ = –3.01 eV), the electronegativity (χ = 3.01 eV), chemical hardness (η = 2.30), chemical softness (σ = 0.22), and the electrophilicity index (ω = 41.18). If a molecule has a large HOMO–LUMO energy gap, it can be considered chemically hard with high polarizability and low chemical reactivity. The relatively large HOMO–LUMO energy gap (4.60 eV) and the corresponding chemical hardness value (η = 2.30 eV) indicate that the title compound is chemically hard to moderately hard, with low chemical reactivity and good kinetic stability. The calculated softness (σ = 0.22 eV^{-1}) further supports its limited charge-transfer capability.

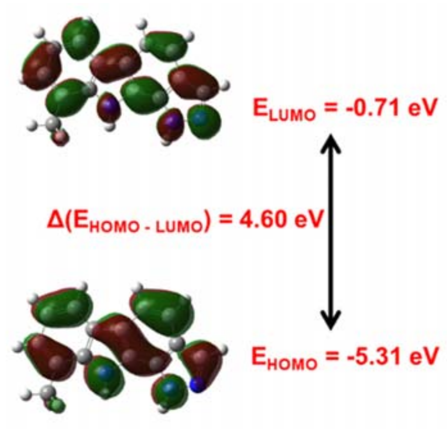

Figure S3

The HOMO to the LUMO energy level diagram.

Electrostatic Potential (ESP) maps help to visualize the charge distribution and other charge related properties of molecules. ESP maps are obtained by the combination of electron densities and electrostatic potential. The electrostatic potential is the energy of interaction of a point positive charge (an electrophile) with the nuclei and electrons. We have positive electrostatic potential and negative electrostatic potential. A positive value shows their areas that are prone to nucleophilic attack, and a negative value suggests their areas that are prone to electrophilic attack. **Figure S4** is MEP of the title molecule with colours scaled from –7.710 10^{-2} (deepest red) to 7.710 10^{-2} (deepest blue), while the intermediary colours are showing the intermediary electrostatic potentials. In the current molecule, the blue regions correspond to areas of positive electrostatic potential, indicating electron-deficient sites. In the title molecule, pronounced positive potential is localized around the hydrogen atoms H26 and H28, which are bonded to the electronegative nitrogen atoms N25 and N27, respectively. These regions are therefore electrophilic in nature and can act as hydrogen-bond donor sites, making them susceptible to interaction with nucleophilic species.

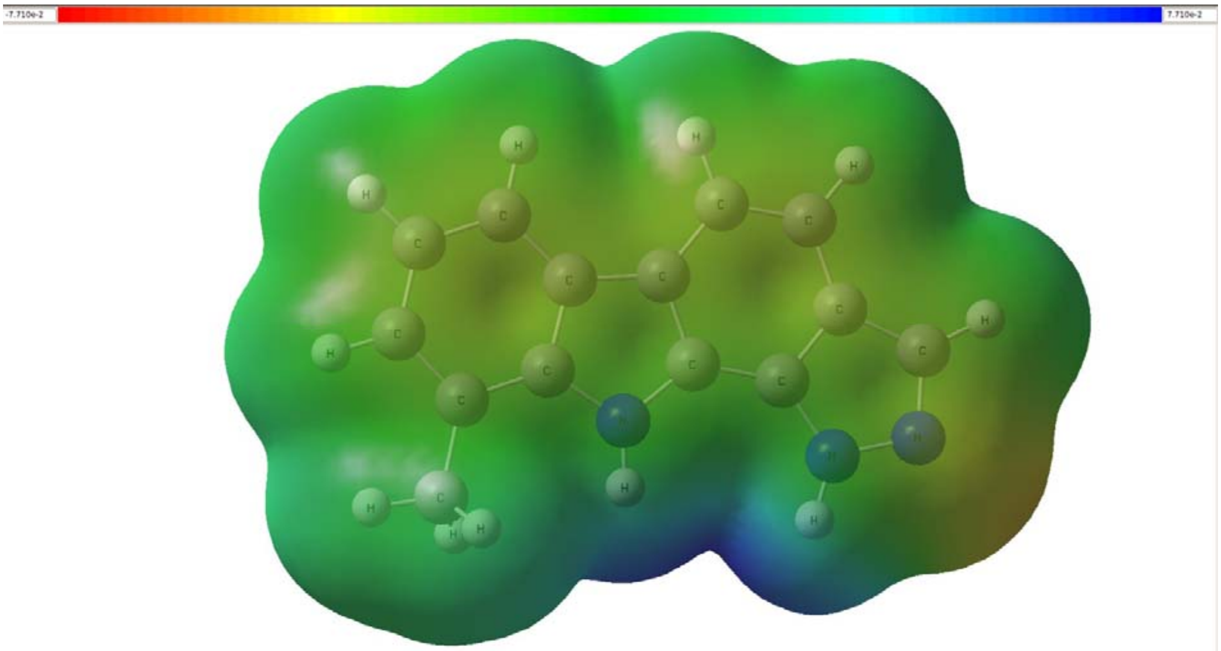

Figure S4

Electrostatic potential map.

S2. Molecular Docking studies:

Initially, we optimized the ligand molecule with the B3LYP/6–31G(d,p) level of theory, and the optimized structure was used for docking analysis. We obtained the target Human Kinase CK2 Protein (PDB ID: 3OWJ) from the Protein Data Bank (https://www.rcsb.org/). The water molecules and ligand in

the target structure were removed. Both ligand and target structures were prepared with Dockamon–PyRx 1.2 software (Dallakyan & Olson, 2014). The best binding pose for the title compound was predicted using the Dockamon–PyRx 1.2 software, as shown in **Figure S5 (a)**. The docking score is –10.4 kcal/mol. The binding score suggests that the compound displays excellent binding affinity against the target Human Kinase CK2 Protein (PDB ID: 3OWJ). The protein-ligand interactions were obtained using the Discovery Studio Visualizer (DSV) 2025 (Discovery Studio Visualizer. Dassault Systèmes, San Diego, 2025. http://www.3dsbiovia.com/) software, which could be easily seen from **Figure S5 (b)**. The above study was performed to evaluate the ligand's potential as a biologically active molecule. CK2 was chosen because it is a clinically relevant, cancer–associated kinase and inhibiting it may lead to the development of new anticancer agents. The strong docking score (–10.4 kcal/mol) suggests that our compound could be a promising CK2 inhibitor.

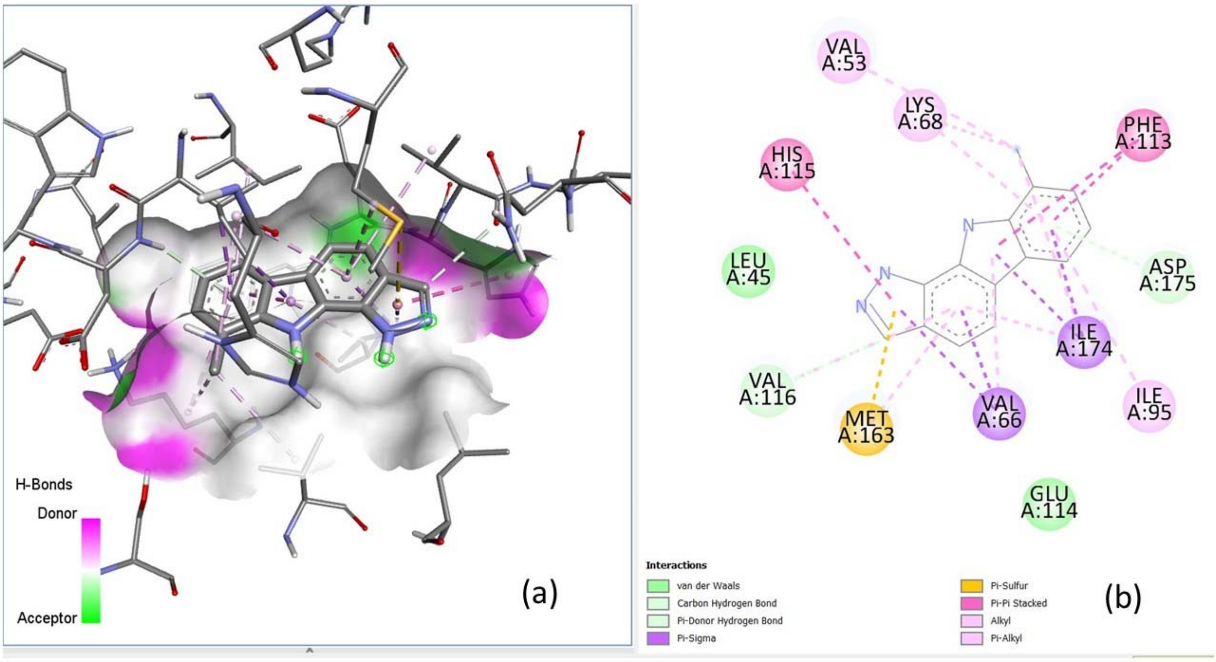

Figure S5

The molecular docking result of the title compound with 3OWJ protein, the surface around (a) and two-dimensional form (b).
